# Expression of polycomb protein BMI‐1 maintains the plasticity of basal bronchial epithelial cells

**DOI:** 10.14814/phy2.12847

**Published:** 2016-08-24

**Authors:** Elizabeth Torr, Meg Heath, Maureen Mee, Dominick Shaw, Tyson V. Sharp, Ian Sayers

**Affiliations:** ^1^ Division of Respiratory MedicineQueens Medical CentreUniversity of NottinghamNottinghamUnited Kingdom; ^2^ Cytogenetics UnitNottingham City HospitalHucknall RoadNottinghamUnited Kingdom; ^3^ School of Life SciencesQueens Medical CentreUniversity of NottinghamNottinghamUnited Kingdom; ^4^ Centre for Molecular OncologyBarts Cancer InstituteQueen MaryUniversity of LondonLondonUnited Kingdom

**Keywords:** BMI‐1, bronchial epithelial cells, lifespan, plasticity

## Abstract

The airway epithelium is altered in respiratory disease and is thought to contribute to disease etiology. A caveat to disease research is that the technique of isolation of bronchial epithelial cells from patients is invasive and cells have a limited lifespan. The aim of this study was to extensively characterize the plasticity of primary human bronchial epithelial cells that have been engineered to delay cell senescence including the ability of these cells to differentiate. Cells were engineered to express BMI‐1 or hTERT using viral vector systems. Cells were characterized at passage (p) early (p5), mid (p10), and late (p15) stage for: BMI‐1, p16, and CK14 protein expression, viability and the ability to differentiate at air–liquid interface (ALI), using a range of techniques including immunohistochemistry (IHC), immunofluorescence (IF), transepithelial electrical resistance (TEER), scanning electron microscopy (SEM), MUC5AC and beta tubulin (BTUB) staining. BMI‐1‐expressing cells maintained elevated levels of the BMI‐1 protein and the epithelial marker CK14 and showed a suppression of p16. BMI‐1‐expressing cells had a viability advantage, differentiated at ALI, and had a normal karyotype. In contrast, hTERT‐expressing cells had a reduced viability, showed limited differentiation, and had an abnormal karyotype. We therefore provide extensive characterization of the plasticity of BMI‐1 expressing cells in the context of the ALI model. These cells retain properties of wild‐type cells and may be useful to characterize respiratory disease mechanisms in vitro over sustained periods.

## Introduction

The airway epithelium acts as the critical interface between the environment and organ physiology. It acts as a barrier to potential pathogens and extraneous particles and helps regulate host defense mechanisms, including the inflammation process. Under normal conditions, the bronchial epithelium is composed of ciliated columnar, mucus‐secreting goblet and Clara cells that secrete surfactant. There is accumulating evidence that the airway epithelium is intrinsically different in airway diseases such as asthma and chronic obstructive pulmonary disease (COPD) if compared with the normal type. For example, bronchial epithelial cells isolated from asthma patients and cultured in vitro have: (1) defective repair mechanisms (Kicic et al. 2010), (2) altered barrier properties including reduced tight junction protein expression and formation (Xiao et al. 2011), (3) elevated expression of proremodeling factors (Lopez‐Guisa et al. 2012), and (4) when differentiated have increased numbers of goblet and reduced numbers of ciliated cells (Parker et al. 2010; Gras et al. 2012). Similarly, genetic studies have identified a large number of asthma susceptibility genes that are differentially expressed in the airway epithelium in asthma including interleukin 33 (IL33) and thymic stromal lymphopoietin (TSLP). This led to the hypothesis that the airway epithelium contributes to disease etiology. Therefore, the airway epithelium represents an attractive therapeutic target in asthma. However, more research tools and cellular assay systems are needed to rapidly move this area of important research forward.

The most common approach to study bronchial epithelial cells from healthy controls and patients with respiratory disease is to isolate cells using bronchoscopic brush technique (Kelsen et al. 1992) and then culture the cells as basal cell monolayers or using air–liquid interface (ALI) differentiation (Stewart et al. 2012a,b). ALI cells form an epithelial barrier that closely resembles the in vivo architecture of the airway epithelium and is composed of basal, goblet, and ciliated cells allowing data to be generated in a more physiological context. However, the bronchoscopic procedure is invasive with risk to the individual, especially in people with severe asthma. Moreover, adequate numbers of cells are hardly collected. Similarly, once isolated and grown in vitro, the primary airway epithelial cells have a limited lifespan (i.e., the point at which the cells stop proliferating, typically 4–5 passages). This is often too short to obtain key experimental data. Importantly, after p4 bronchial epithelial cells begin to deviate in their morphology/phenotype, lose their plasticity required for the differentiation at ALI. These factors make the identification and careful characterization of a method to delay cell senescence while maintaining the plasticity of the cells highly desirable. One approach to delay cell senescence is the suppression of cyclin‐dependent kinase inhibitor, p16(Ink4a), a tumor suppressor that induces a G1 cell cycle arrest. B‐cell‐specific Moloney murine leukemia virus integration site 1 (BMI‐1) is a polycomb protein thought to repress p16(Ink4a) expression and has previously been shown to delay cell senescence (Vonlanthen et al. 2001).

In this study, we set out to engineer primary human bronchial epithelial cells to express different levels of BMI‐1 via alternative promoter constructs and extensively characterize the cells for effects on viability and plasticity such as the ability to differentiate at ALI. This was assessed using a range of techniques including immunohistochemistry (IHC), immunofluorescence (IF), transepithelial electrical resistance (TEER), and scanning electron microscopy (SEM). Importantly, we also investigated genome stability/integrity through effects on karyotype. Both wild‐type early passage cells and cells engineered to express hTERT (as a commonly used immortalization technique) were included in these analyses. We demonstrate the BMI‐1‐engineered cells assessed at early (p5), mid (p10), and late (p15) passage produce elevated levels of BMI‐1 expression with associated suppression of p16. These cells retain both viability and differentiation potential of wild‐type cells while importantly not demonstrating the changes in cell karyotype. These features were apparent across all donor cell populations engineered to express the elevated levels of BMI‐1. These data are in contrast to hTERT cells that were no longer viable and did not differentiate at mid passage and demonstrated gross chromosomal abnormalities. Therefore, BMI‐1‐engineered bronchial epithelial cells delayed cell senescence and retained their plasticity including the ability to differentiate at ALI in a comparative manner to low passage unmodified cells.

## Materials and Methods

### BMI‐1 plasmid construction

Human BMI‐1 full‐length cDNA was originally obtained from Geneservice^™^ Mammalian Gene Collection (MGC) Clone, amplified and cloned into pLVX‐Puro (Clontech) using XhoI/BamHI restriction sites and into pFLRu‐FH using MluI/BamHI restriction sites, using standard molecular biology techniques. The retroviral vector pWZL‐BLAST‐FLAG‐HA‐hTERT and corresponding empty vector backbone were purchased from Addgene (Maida et al. 2009). All vectors were sequence verified.

### Packaging of lentivirus and retrovirus

HEK293T cells were seeded at 2 × 10^6^ cells per 10‐cm diameter dish and were cultured in DMEM, 10% FCS, 1% penicillin/streptomycin overnight. For lentiviral production, the cells were transfected with a mix containing an 8:1 ratio of packaging: envelope vectors, that is, 4.4 *μ*g of pCMV delta R8.9, 0.6 *μ*g of pCMV‐VSV‐G (McKay et al. 2011), and 5 *μ*g of the appropriate lentiviral vector (empty or BMI‐1 insert) with 36 *μ*L of TransIT‐LT1 (Geneflow). For retroviral production, the cells were transfected with 5 *μ*g of pKAT (Addgene) and 5 *μ*g of retroviral vector (empty or with hTERT insert) and 36 *μ*L of TransIT‐LT1 (Geneflow). Cells were grown for 48–72 h and the supernatant containing packaged virus was harvested and ultracentrifuged (48,384 *g* for 3.5 h at 4°C) to produce a concentrated viral stock which was stored at ‐80°C.

### Primary bronchial epithelial cell culture and air liquid interface

Normal Human Bronchial Epithelial Cells (NHBEC) were purchased from Lonza, (Wokingham, UK). Donor 1 cells were isolated from a 43‐year‐old Caucasian male with no history of smoking; Donor 2 cells were isolated from a 56‐year‐old Caucasian male smoker. NHBEC were grown in a growth factor‐supplemented medium (BEGM) (Lonza) and differentiated at ALI in bronchial epithelial differentiation medium (BEDM), according to our previously published methods (Stewart et al. 2012a,b). BEDM is composed of 50:50 Dulbecco's Modified Eagle's Medium (DMEM, Sigma‐Aldrich, Dorset, UK):BEBM with Lonza singlequots, excluding triiodo‐L‐thyronine and retinoic acid, but including GA‐1000 (Gentamicin and Amphotericin‐B). BEDM is supplemented with 50 nmol/L retinoic acid, added at time of use. All cells were cultured on 6.5‐mm polyester Transwell inserts with a pore size of 0.4 *μ*m (Corning Life Sciences, Amsterdam, The Netherlands). Cells were plated at 30,000 cells per insert in an appropriate medium. When confluent (~3 days), cells were raised to ALI. Medium was replaced and the apical face washed with media every 48 h. Cells were fixed for immunostaining, histology sectioning, and scanning electron microscopy after 28 days at ALI.

### Generation of genetically modified NHBEC

Passage 2 NHBEC were plated in a 6‐well plate at 5 × 10^4^ cells per well and grown overnight. These were purchased heterogeneous NHBEC and not clonal cells. The media was replaced with 800 *μ*L of BEGM with 2 *μ*g/mL of polybrene (Hexadimethrine bromide H9268 Sigma) and 6.25 *μ*L of lentivirus or 12.5 *μ*L of retrovirus stock solutions (minimal concentrations to give >90% transfection determined empirically). The plates were incubated at 37°C for 6.5 h with gentle rocking of the plate every 30 min for the first 2 h. The viral mixture was removed and replaced with BEGM. After 72 h, selection media was added to the wells with 300 ng/mL puromycin for the lentiviral‐infected cells and 600 ng/mL blasticidin for the retroviral cells. Cells with antibiotic resistance after 6 days of selection were considered to have been stably infected with the appropriate virus expressing BMI‐1 or hTERT.

### Western blotting

A total of 2 × 10^5^ cells were lysed in SDS loading buffer, heat denatured, and proteins were separated by electrophoresis using 12% SDS resolving gels. The proteins were transferred to nitrocellulose and probed for BMI‐1, p16, and *β* actin expression using mouse anti‐p16 (Millipore MAB4133; Watford, UK) at 1 in 1000 dilution (1 *μ*g/mL), mouse anti‐BMI‐1 (Millipore Clone F6 Cat No 05‐637) primary antibody at 1 in 1000 dilution, and rabbit polyclonal anti‐*β* actin (Abcam ab8227, lot 712923, 0.65 mg/mL) primary antibody at 1 in 5000 dilution. Secondary antibodies were used at 1 in 10000 dilutions and consisted of goat anti‐mouse HRP (Jackson Immuno 115‐035‐062) and goat polyclonal anti‐rabbit HRP (Sigma A0545). ECL reagent was used to visualize proteins as directed by the manufacturer (GE Healthcare RPN2209; GE Healthcare, Amersham, UK).

### Immunofluorescence

ALI‐cultured cells were fixed in situ on inserts and transferred to the glass slides for visualization. Cells were fixed using 4% formaldehyde and blocked/permeabilized with PBS, 10% goat serum, 1% BSA, and 0.15% Triton‐X. Cells were incubated with appropriate primary antibodies at 4°C overnight, and FITC labeled secondary for 1 h at room temperature before mounting in HardSet DAPI (Vector Labs). These methods were as previously described (Stewart et al. 2012b) with the addition of BMI‐1 (Millipore Clone F6) and CK14 (Chemicon MAB3232) antibodies to the panel. Cells were visualized using the Zeiss spinning disk confocal microscope and Volocity software (PerkinElmer, Cambridge, UK).

### Cell viability

A total of 2.5 × 10^3^ cells were plated in quadruplicate wells of a 96‐well plate in 200 *μ*L BEGM + GA and incubated for 96–120 h before analysis. 3‐(4,5‐dimethylthiazol‐2‐yl)‐2,5‐diphenyltetrazolium bromide (MTT) at 500 *μ*g/mL in BEGM was added to the wells for 4 h before termination of the experiment. The media was then replaced with 200 *μ*L isopropanol and incubated for 10 min to lyse the cells. The plate was read at 570 nm (with a background subtraction of 690 nm) using a Flexstation (Molecular Devices, Wokingham, UK).

### Transepithelial electrical resistance

Transepithelial electrical resistance (TEER) was measured in differentiating cells using an EVOM2 epithelial volt‐ohm meter (World precision Instruments UK, Stevenage) to indicate development of tight junctions as described previously (Stewart et al. 2012b). Briefly, medium was aspirated and replaced with 1 mL growth medium in the basolateral and 0.5 mL growth medium in the apical compartment. Cultures were equilibrated in the incubator for 30 min before TEER measurement. Apical medium was then aspirated to restore ALI. TEER values of insert and medium alone wells was subtracted from the measured TEER and Ωcm^2^ was calculated by multiplying by the insert area. For comparison across cell lines and passage, we calculated area under curve using TEER data from days 1–28 using GraphPad Prism 6.02 for Windows (GraphPad Software, San Diego, CA).

### Karyotyping

Cells in active growth phase were treated with demecolcine solution (Sigma) at 400 ng/mL for 4 h before harvesting, swelling (hypotonic solution [0.056 mol/L KCL]), and fixing in methanol/glacial acetic acid. The cells were fixed further two times before slides were produced and aged, then banded using trypsin and Giemsa staining before imaging and analyses (completed by Cytogenetics Unit, City Hospital, Nottingham, UK).

### Histological analyses

Fixed transwells were paraffin embedded, sectioned, and then stained with hematoxylin and eosin (H & E) or alcian blue using standard approaches.

### Scanning electron microscopy

Transwells at day 28 ALI were washed with BEGM media to remove any mucous and then fixed with 3% glutaraldehyde in 0.1 mol/L sodium cacodylate buffer for a minimum of 24 h. The wells were washed three times with 0.1 mol/L phosphate/cacodylate buffer and post fixed for 1 h at room temperature with 1% aqueous osmium tetroxide. The samples were washed with distilled water and then dehydrated using a graded series of ethanol washes (50, 70, 90, and 100%, respectively). Samples remained submerged in 100% ethanol, processed when they were dried, and coated with gold particles. The samples were then imaged at low and high magnification using a scanning electron microscope (model JSM‐35; JOEL).

### Telomerase activity assay

TRAPeze XL Telomerase detection kit was used as directed by the manufacturer to determine hTERT activity (Millipore S7707). A total of 1 × 10^5^ cells were suspended in 200 *μ*L of CHAPS lysis buffer and incubated on ice for 30 min. The positive cells (1 × 10^6^, provided by the kit) were treated in the same way. The samples were then centrifuged at 120,000g for 20 min at 4°C. PCR tubes containing 10 *μ*L 5X TRAPeze XL reaction mix, 0.4 *μ*L Taq polymerase (5units/*μ*L), 37.6 *μ*L dH_2_O, and 2 *μ*L of cell extract, positive template or buffer for the control samples were run at 30°C for 30 mins followed by 36 cycles of 94°C for 30 sec, 59°C for 30 sec, and 72°C for 1 min. This was followed by 72°C for 3 min, 55°C for 25 min, and 4°C hold. The samples were then diluted in 150 *μ*L of buffer (10 mmol/L Tris‐HCl pH 7.4, 0.15 mol/L NaCl, 2 mmol/L MgCl_2_) and fluorescent signals were read at excitation 485 nm and emission 535 nm and excitation 585 nm and emission 620 nm on the Flexstation (Molecular Devices).

### Statistical analyses

GraphPad Prism 6.02 for Windows was used for statistical analyses. Quantitative data were analyzed using Student's *t* test or ANOVA with Dunnett's multiple comparison test. A *P* <0.05 was considered significant.

## Results

### Cell generation

We specifically chose two lentiviral vectors with differing promoters: Cytomegalovirus (CMV) in pLVX‐Puro or human ubiquitin C (UbiC)) in pFLRu‐FH to express BMI‐1 in human bronchial epithelial cells with the hypothesis that the CMV promoter may lead to greater BMI‐1 overexpression than the UbiC promoter (Qin et al. 2010). Lentiviruses were used to transduce passage 2 NHBECs from two donors. This protocol therefore generated four independent cell populations expressing recombinant BMI‐1 to evaluate the effect of BMI‐1 on cell plasticity. The transduction efficiency was >95%, as indicated by limited cell death following antibiotic selection and the pWZL‐based constructs gave similar findings (data not shown). Cell populations generated were followed up for ~12 months with focused analyses presented for (1) Early passage (infection passage 3/actual passage 6), (2) Mid passage (infection passage 5–7/actual passage 8–10), and (3) Late passage (infection passage 8–12/actual passage 11–15). Finally, a subset of analyses was completed using extended passage cells (infection passage 17/actual passage 20). It is important to note that we arbitrarily designated these early, mid, and late passage definitions based on passage 20 being the maximum achieved. NHBECs transduced with lentivirus containing plasmid vector that did not contain the BMI‐1 expression cassette, that is, empty vectors, stopped growing at mid passage. Similarly, for the cells containing the pWZL constructs including hTERT insert cells were not viable at mid passage, although there were donor differences in these cell senescence times.

### NHBEC‐BMI‐1 cell populations maintain elevated levels of BMI‐1 expression and feature p16 suppression during prolonged culture

**Figure 1:**
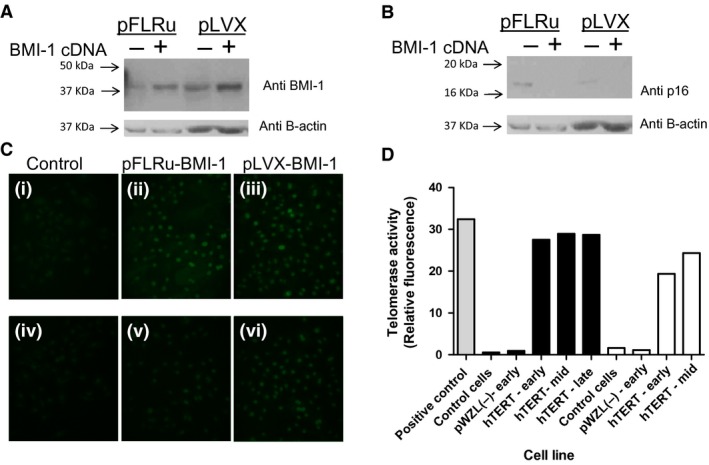
BMI‐1 epithelial cell populations express elevated levels of BMI‐1 protein and show suppression of p16 protein levels. (A) Early passage‐engineered cells express elevated levels of BMI‐1 compared to empty vector control cell population as determined by Western blot and (B) have suppressed levels of p16, (C) BMI‐1 immunofluorescence analyses for wild‐type cells donor cells (control) show moderate BMI1 levels, whereas BMI‐1‐engineered late passage cells show elevated levels in both vector‐based approaches, Donor 1 (i–iii) and Donor 2 (iv–vi)). (D) hTERT activity is elevated in pWZL‐hTERT cell population in both donors compared to wild‐type cells (control) and control vector cells (individual experiment for each donor). Donor 1 (fill) and donor 2 (no fill). Positive control is provided by the assay kit. *Note late passage data not available for pWZL‐hTERT donor 2 as this cell population stopped growing.

Both the pFLRu‐BMI‐1(UbiC promoter) and pLVX‐BMI‐1 (CMV promoter)‐engineered cells demonstrated elevated BMI‐1 expression as evidenced by Western blotting in comparison with control vectors without insert at early passage (Fig. 1A). Importantly, the elevated level of BMI‐1 observed in these cell populations was accompanied by a decrease in the level of CDK inhibitor p16 (Fig. 1B). Similarly, BMI‐1 immunofluorescence for wild‐type cells (control) at passage 3 showed weak BMI‐1 protein expression in comparison with high nuclear BMI‐1 expression in engineered late passage cells for both donors demonstrating robust elevated BMI‐1 through to late passage (Fig. 1C). Early and mid passage cells showed similar elevated BMI‐1 expression as determined by immunofluorescence (data not shown). In order to confirm that genetic modification of cells to overexpress hTERT translated to increased hTERT enzymatic activity, we determined telomerase activity in wild‐type cells, cells transfected with empty vector, and those cells engineered to express hTERT. hTERT activity was elevated in the hTERT‐expressing cell populations in both donors across early‐mid‐late passages as anticipated (Fig. 1D).

### NHBEC‐BMI‐1 cell populations have extended viability

**Figure 2:**
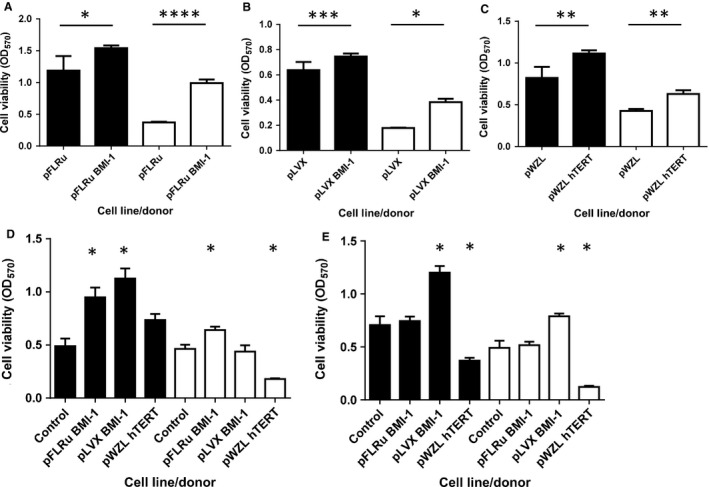
BMI‐1 epithelial cell populations have an extended lifespan with viability increased compared to wild‐type bronchial epithelial cells. Viability (MTT assay) was determined for early passage cells at (A) pFLRu/96 h, (B) pLVX/96 h, and (C) pWZL/120 h demonstrating that the insertion of BMI1 or hTERT leads to increased viability in Donor 1 (solid bars) and Donor 2 (open bars), *P < 0.05, **P < 0.005, ***P < 0.0005, ****P < 0.00005. (D) Mid passage‐engineered cells show alterations in viability compared to wild‐type cells assayed for 96 h. (E) Late passage viability (assayed for 120 h). *note donor 2 hTERT cell population is mid passage (maximum passage achieved).

In order to investigate the effect of the genetic modifications on cell viability, we used the MTT assay as described (Portelli et al. 2014). Early passage cells containing either BMI‐1 vector or pWZL‐hTERT showed increased viability in both donors over 96 or 120 h (Fig. 2A, B and C, *P* < 0.05). Similarly, mid passage cells showed alterations in viability compared to wild‐type cells over 96 h (empty vector controls were no longer viable at this stage). For donor 1, both BMI‐1 cell populations increased viability compared to wild‐type cells (ANOVA, post hoc *P* < 0.05), whereas hTERT‐engineered cells did not show altered viability compared to wild‐type control. For donor 2, pFLRu‐BMI‐1 significantly increased cell viability and hTERT reduced viability when compared to wild‐type control (ANOVA, post hoc *P* < 0.05) with pLVX‐BMI‐1 cell viability showing no difference (Fig. 2D). In a similar analysis of late passage cells (assayed over 120 h), both donors showed pLVX‐BMI‐1 significantly increased viability, whereas hTERT cells had a decreased viability (Fig. 2E, ANOVA, post hoc *P* < 0.05). Late passage pFLRu‐BMI‐1 cells showed no differences in viability compared to wild‐type control cells. These data suggest that late passage NHBECs transduced with the pLVX‐BMI‐1 lentivirus are the only cell populations to maintain high viability.

### NHBEC‐BMI‐1 cell populations maintain epithelial cell marker expression over prolonged lifespan

**Figure 3:**
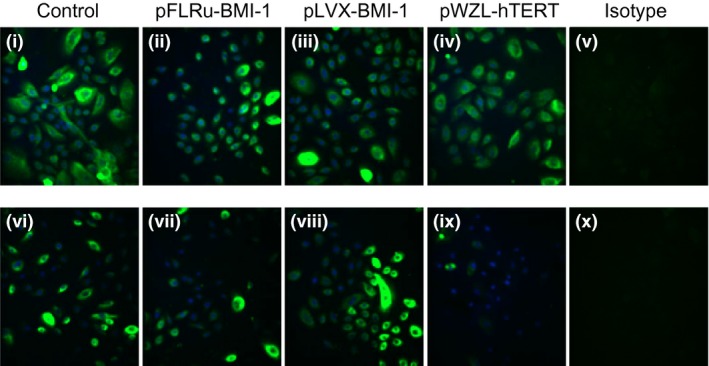
BMI‐1 epithelial cell populations retain epithelial cell marker expression over extended passage. Immunofluorescence for cytokeratin 14 confirmed epithelial lineage in wild‐type cells (Control) and for late passage engineered cells for Donor 1 (i–iv) and Donor 2 (vi–ix). Note pWZL‐hTERT in donor 2, mid passage was the maximum passage achieved.

In order to begin to characterize the cell phenotype in the genetically modified cells, we examined the expression of the epithelial lineage marker, cytokeratin (CK) 14. For both donors, a very similar level and distribution of CK14 was observed in wild‐type cells at passage 3 and late passage cells for both BMI‐1 vectors using immunofluorescence (Fig. 3). Similarly, donor 1 hTERT‐expressing cells showed comparable expression, however, for donor 2 pWZL‐hTERT, the cells did not reach late passage and infection passage 8 (passage 10) was the maximum achieved with limited CK14 expression observed.

### ALI differentiated NHBEC‐BMI‐1 cell populations show comparative morphology to nonmodified wild‐type cells

**Figure 4:**
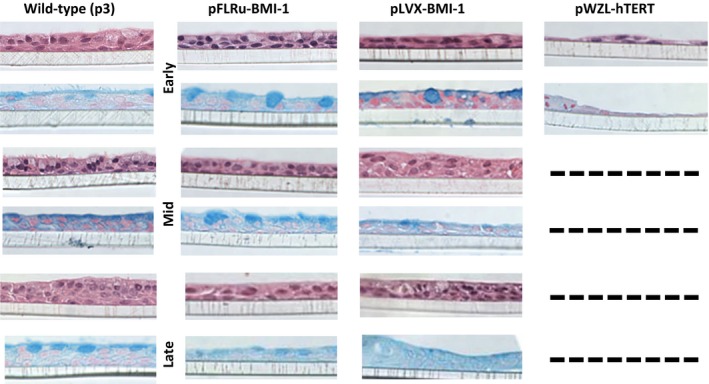
BMI‐1 epithelial cell populations retain the ability to differentiate at air–liquid interface and show comparative cell composition and morphology to wild‐type cells. Sections of air–liquid interface cultures stained with hematoxylin and eosin or alcian blue stains. Early, mid, and late passage BMI‐1‐expressing cells maintain morphology demonstrating a pseudostratified layer with evidence of differentiation into mucus‐producing cells similar to matched wild‐type cells (passage 3). Wild‐type cells were included in each of the early, mid, and late time point experiments, hence, three panels. hTERT cell populations were not available for all passages as these did not form differentiated layers and the cells clumps did not adhere to transwells, hence, after fixing these cells became detached. Data shown for Donor 2, Donor 1 showed similar results.

To compare the morphology of genetically modified cells to low passage (p3) wild‐type cells grown at air–liquid interface, we examined sections stained with H & E and alcian blue to detect mucins. Wild‐type NHBECs generated a pseudostratifed epithelial layer upon ALI showing similarities to that observed in situ including mucin staining at the apical side and the presence of cilia (Fig. 4). Significantly, early, mid, and late passage BMI‐1‐expressing cells from both lenti‐vector constructs maintained the ability to differentiate and showed the classical pseudostratified epithelial layer in this model, however, late passage BMI‐1 cells had more diffuse mucin staining (Fig. 4). In contrast, hTERT‐expressing cells failed to show a differentiated phenotype even at early passage and in mid and late passage, it was not possible to generate sections as the cells did not grow and adhere to the transwell.

### NHBEC‐BMI‐1 cell populations retain the ability to differentiate into multiple cell types at air liquid interface

**Figure 5:**
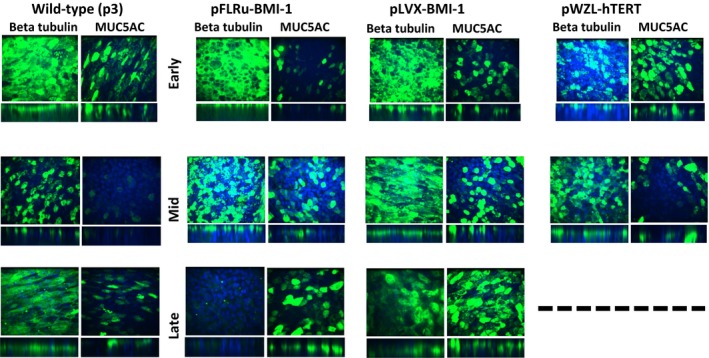
BMI‐1 epithelial cell populations retain the ability to differentiate at air–liquid interface and stain positively for goblet and ciliated cell markers. Immunofluorescence including z‐stacks of markers for goblet cells (MUC5AC) and ciliated cells (beta tubulin IV) generated in early, mid, and late passage BMI‐1 or hTERT‐expressing cells with wild‐type control cells (p3). Wild‐type cells were included in each of the early, mid, and late time point experiments, hence, three panels. Data from donor 1 and representative of both donors, however, donor 2 hTERT‐expressing cells did not provide robust ALI cultures post early passage. Note some data were available for hTERT mid passage cells (in contrast to Fig. 4) due to the gentler processing of the samples for IF. ALI, air–liquid interface; IF, immunofluorescence

In order to further characterize the differentiated phenotype of wild‐type and genetically modified cells, we used immunofluorescence for MUC5AC which is a marker of goblet cells and beta tubulin, which is a marker of ciliated cells. These data show that early, mid, and late passage BMI‐1‐expressing cells maintain differentiation potential and generate a differentiated layer composed of multiple cell types comparable to wild‐type control cells from the same experiments (Fig. 5). As anticipated in the z‐stack images, both the beta tubulin and MUC5AC staining is particularly apical. hTERT‐expressing cells maintain these features until cell senescence for this donor.

### NHBEC‐BMI‐1 cell populations retain the ability to differentiate and form an epithelial barrier

**Figure 6:**
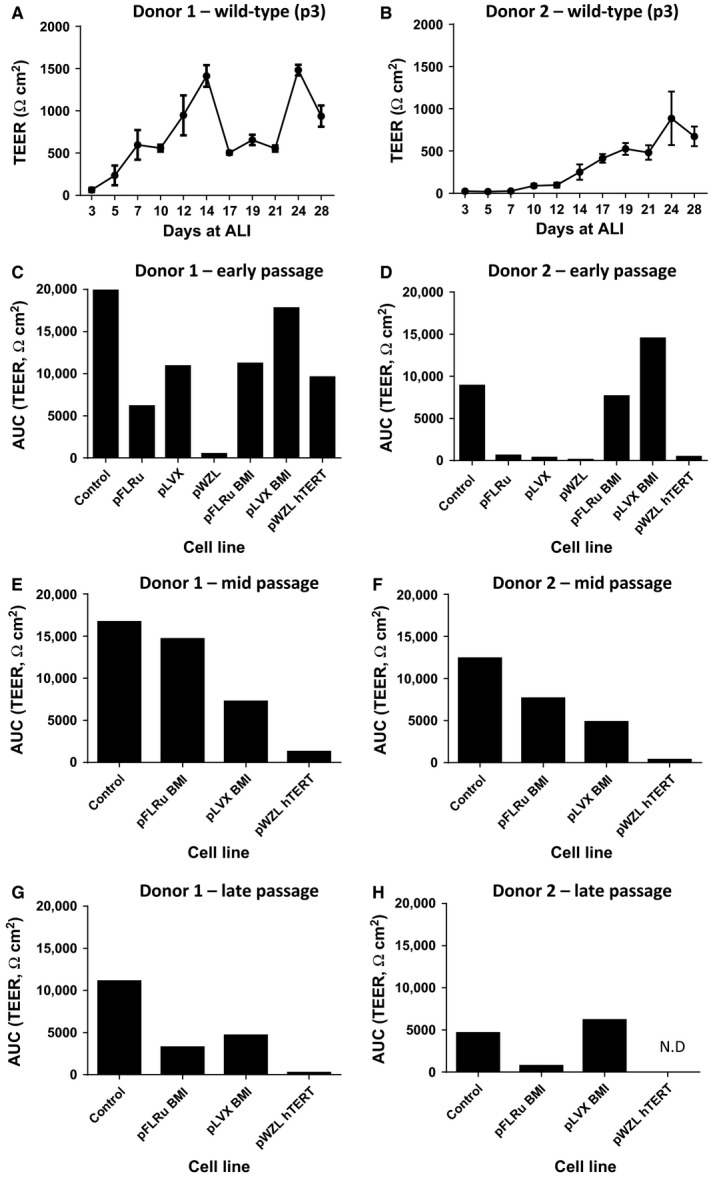
BMI‐1 epithelial cell populations retain the ability to differentiate at air–liquid interface and develop transepithelial electrical resistance. Representative TEER traces for (A) donor 1 and (B) donor 2 wild‐type cells at passage 3 demonstrating increasing in TEER during the ALI differentiation. Area under the curve (AUC) analyses of TEER for early passage‐engineered cells for (C) donor 1 and (D) donor 2. Mid passage TEER (AUC) for (E) donor 1 and (F) donor 2 and late passage TEER (AUC) for (G) donor 1 and (H) donor 2. Empty vector control cells failed to grow after early passage stage and so are not present on graphs (E–H). Data shows a single experiment for both donors at each passage. ALI, air–liquid interface; TEER, transepithelial electrical resistance.

One of the hallmarks of the ALI model is the development of tight junctions between the epithelial cells forming a barrier. A surrogate measure of this is the development of TEER. Therefore, we examined this outcome in both wild‐type and genetically modified cells. Both donors demonstrated the capacity to develop TEER as shown by the wild‐type cells (passage 3) time course data (Fig. 6A and B). In Figure 6A, a decrease in TEER was observed between days 14–21 which we have observed for other primary human cell donors. Similar plots were generated for early, mid, and late passage‐engineered cells and to facilitate multiple comparisons area under the curve values were generated. At early passage donor 1 cells, all developed a TEER except for the pWZL empty vector cell population (Fig. 6C). The level of TEER was variable with pLVX‐BMI‐1 showing the most comparable to wild‐type control levels (Fig. 6C). Donor 2 early passage data demonstrated that only the BMI‐1‐containing cells were capable of developing a TEER and in the case of the pLVX‐BMI‐1, this was higher than the wild‐type control. All empty vector controls and the pWZL‐hTERT did not develop a measurable TEER at passage 6 (Fig. 6D). At mid passage, the empty vector controls were no longer viable and comparison of the engineered cells showed that in both donors, the BMI‐1‐expressing cells demonstrated a measurable TEER, albeit lower than wild‐type, whereas the hTERT‐engineered cells did not (Fig. 6E and F). At late passage, again the BMI‐1‐expressing cells demonstrated a TEER but at a reduced level compared to wild‐type cells (Fig. 6G and H). Across the passage stages, there was a gradual loss of TEER development in the BMI‐1 cell lines when compared to the wild‐type cells (100%) at each stage, for example, Donor 1 pFLRu‐BMI‐1: 56.5%, 28.1%, and 29.1% (of wild‐type control, 100%) across early, mid, and late passages. Similarly, Donor 1 pLVX‐BMI‐1: 89.5%, 43.5%, 42.1% (of wild‐type control, 100%) across early, mid, and late passages. Donor 2 data were more heterogeneous but showed a similar trend. These data demonstrated that the BMI‐1‐expressing cells maintain plasticity, differentiate at ALI, and develop TEER. However, due to our low number of technical replicates, we were not able to provide more quantitative comparisons of the absolute levels of differentiation, for example, TEER achieved across cell populations and/or passages with certainty.

### NHBEC‐BMI‐1 cell populations retain the ability to differentiate forming ciliated cells at the apical surface

**Figure 7:**
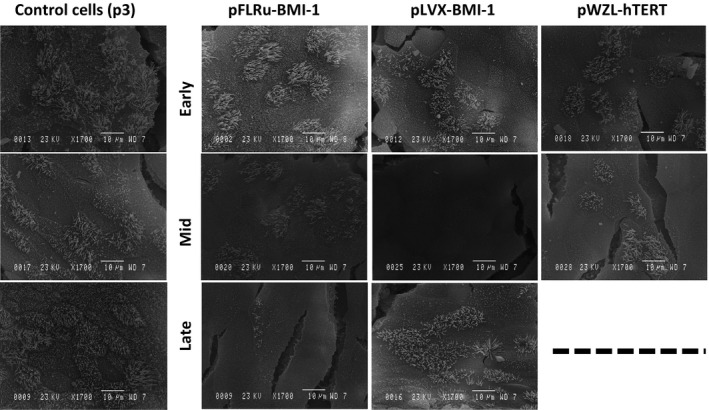
BMI‐1 epithelial cell populations retain the ability to differentiate at air–liquid interface and demonstrate surface cilia in scanning electron microscopy. Wild‐type control cells at passage 3 show evidence of ciliated cells on the surface of air–liquid interface cultures using SEM. Wild‐type cells were included in each of the early, mid, and late time point experiments, hence, three panels. Both BMI‐1‐ and hTERT (when viable)‐expressing cells grown at ALI show some evidence of ciliated cells on the surface. Data taken from donor 1 and representative of both donors were analyzed (×1700 magnification). ALI, air–liquid interface; SEM, scanning electron microscopy.

To further characterize the differentiated epithelial layer observed in the ALI model, we used scanning electron microscopy to visualize the apical surface and examine cilia. These images clearly show cilia projections on the surface of wild‐type cells as we have observed previously (Fig. 7). For genetically engineered cells, cilia were also present including the hTERT genetically modified cells and there was a large degree of heterogeneity. For mid passage pLVX‐BMI‐1 cells, there was potentially a reduced pattern of cilia compared to wild‐type control. Importantly, there was evidence for late passage BMI‐1 cells, particularly the pLVX‐BMI‐1 cells to have ciliated surfaces (Fig. 7).

### NHBEC‐BMI‐1 cell populations maintain a stable genome over extended passage

**Figure 8:**
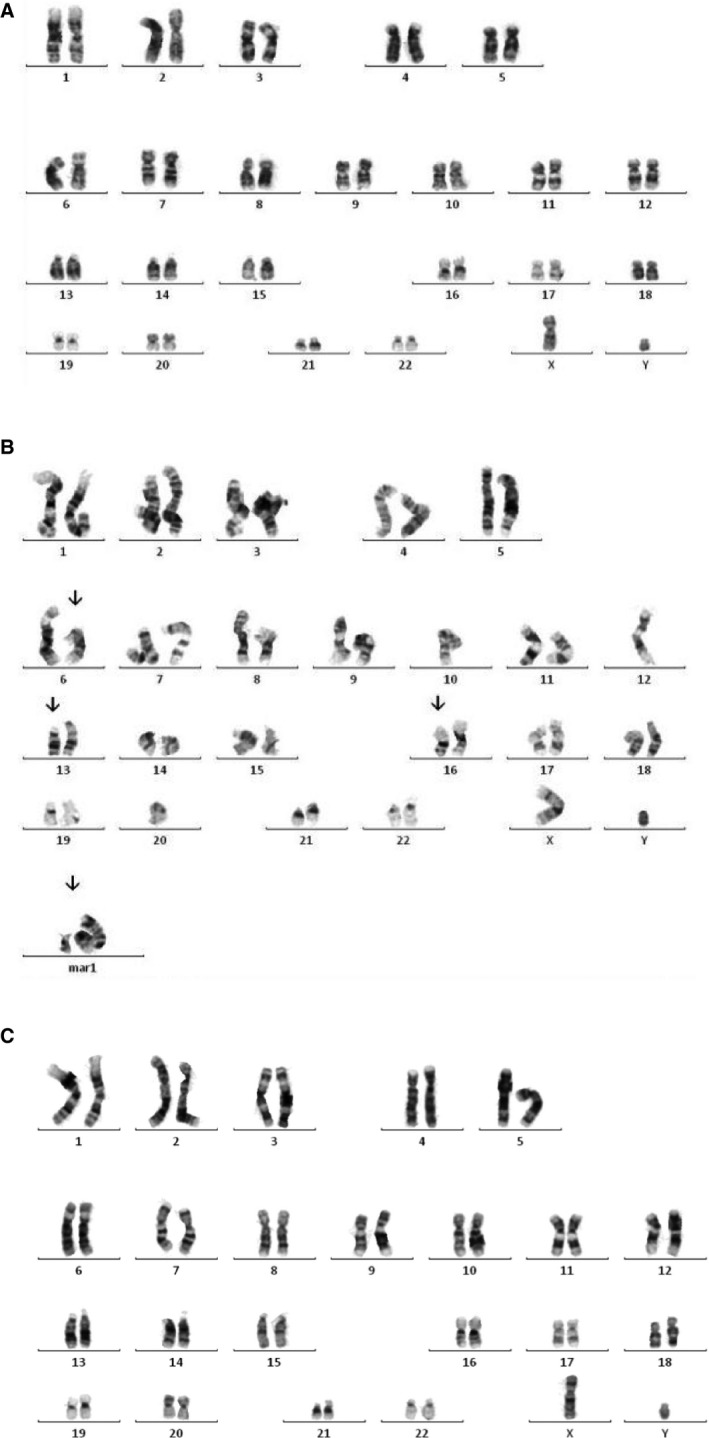
Karyotyping identifies abnormalities in the hTERT not BMI‐1‐engineered cell populations. (A) Representative normal karyotype showing 22 autosomes and X and Y chromosomes (donor 2 passage 3 cells), (B) Abnormal karyotype including alterations on chromosomes 6, 13, and 16 and chromosomal fragments that could not be mapped observed for donor 2 hTERT mid passage cells and (C) Normal karyotype for late passage donor 2 pFLRu‐BMI‐1 cells.

As many approaches to prevent cell senescence have unwanted effects on the genetic information of the cells, we completed karyotyping of wild‐type and genetically modified cells at each passage stage to identify gross changes in chromosomes (>5 megabase rearrangements). For all BMI‐1‐expressing cells assayed at early, mid, and late passage, normal karyotypes were present. In contrast, an abnormal karyotype was observed for donor 2 hTERT‐expressing cells at mid passage suggesting gross alterations on chromosomes 6, 13, and 16 and chromosomal fragments that could not be mapped (Fig. 8).

## Discussion

In this study, we set out to evaluate methods to delay cell senescence while maintaining plasticity/differentiation potential in primary human bronchial epithelial cells. This is important as these cells have a key role to play in respiratory disease warranting further research. However, such primary cells are isolated from patients by invasive procedures and have limited lifespan ex vivo. We have developed two lentiviral systems that enabled us to elevate the expression of polycomb protein BMI‐1 and importantly suppress p16 levels in cells containing these genetic modifications. Extensive characterization over 12 months demonstrated that cells expressing higher levels of BMI‐1 have (1) increased viability, (2) an extended number of cell divisions, (3) maintain basal epithelial morphology, (4) maintain plasticity, that is, the ability to form a differentiated pseudostratified air–liquid interface model, and (5) importantly maintain a normal karyotype. These data suggest that the cell populations generated using BMI‐1 induction maintain cell plasticity over extended periods in the laboratory and may be useful for primary human bronchial epithelial cell research.

To date, either viral oncogenes have been used to prevent cell senescence in human bronchial epithelial cells (Willey et al. 1991; Zeitlin et al. 1991; Viallet et al. 1994) or the introduction of human telomerase reverse transcriptase (hTERT) (Zabner et al. 2003; Piao et al. 2005). These studies have shown some success, however, a major limitation is alterations in karyotype (Piao et al. 2005). Therefore, viral oncogene independent approaches for prevention of cell senescence have been sought, the most favored approach being one with an underlying mechanism that decreases the levels of cyclin‐dependent kinase inhibitor, p16(Ink4a), a tumor suppressor that induces a G1 cell cycle arrest.

BMI‐1 is a polycomb transcriptional repressor protein and is part of the polycomb repressive complex‐1 (PRC1) complex. This complex regulates transcriptional activity at several loci including the Ink4a locus which encodes the tumor suppressor proteins p16(Ink4a) and p14(Arf). BMI‐1 has been extensively characterized in the context of numerous cancers, with both protein and mRNA levels correlating with disease prognosis (Cao et al. 2011). The contribution of BMI‐1 to multiple cancers is thought to be due to its role in self‐renewal and differentiation of stem cells (Lessard and Sauvageau 2003). With these unique properties of regulating cell senescence, BMI‐1 has been the focus of attention particularly in the delay of cell senescence in many cell types, for example, stem cell feeder cells (McKay et al. 2011). Recently, transduction of HBEC with combinations of hTERT and BMI‐1 has generated some promising data (Fulcher et al. 2009). The resultant hTERT/BMI‐1 cell populations continued to divide longer than nonmodified cells, and importantly, at passage 14 and 15 had a diploid karyotype and formed differentiated pseudostratified morphology following ALI culture. Interestingly, BMI‐1 alone was also introduced to bronchial epithelial cells in the study of Fulcher and colleagues and delayed cell senescence, however, these BMI‐1 cell populations were not extensively characterized for plasticity, a hallmark of low passage basal epithelial cells.

In this study, we set out to significantly extend these studies and focus away from the use of hTERT to the use of BMI‐1 alone to delay cell senescence in bronchial epithelial cells and provide more definitive characterization of the plasticity of cells particularly in the context of ALI models. To this end, we developed two new lentiviral systems engineered to express elevated BMI‐1 under the control of CMV or UbiC promoter and a control retroviral system expressing hTERT for comparison. These systems were used to transduce passage 2 normal bronchial epithelial cells from two donors. These genetically engineered cells showed sustained elevation of BMI‐1 as determined by western blot and immunofluorescence and similarly elevated telomerase activity for the hTERT‐engineered cells. As anticipated, the BMI‐1‐expressing cells showed consequent suppression of p16 as observed by several groups (Meng et al. 2010). It is important to note that BMI‐1 is a master regulator of a very large number of genes and gene families of relevance to cell senescence, for example, see a recent study using microarray analyses in BMI‐1 knockout mice cells (Zacharek et al. 2011). In this study, a large number of genes were differentially expressed (elevated) when BMI‐1 was absent which included multiple homeodomain genes and Cdkn2a (p16/p19) and Cdkn2b (p15). In a more recent study using BMI‐1 −/− mouse brain tissue in conjunction with RNA‐seq, >500 genes were shown to be up regulated due to a loss of BMI‐1 including identifying a role for BMI‐1 in TGF‐β/BMP‐ER stress pathways (Gargiulo et al. 2013). Therefore, while our p16 suppression data confirms that our genetic manipulation of BMI‐1 has downstream effects on BMI‐1 responsive networks, it is highly likely that multiple pathways underlie the effects we have observed, for example, p14Arf suppression by BMI‐1 may be highly relevant for apoptosis mechanisms through the mdm2/TP53 pathway.

In the first set of experiments, we demonstrated that early passage BMI‐1 cells have a slightly higher viability as did hTERT cells, however, as the passage number increased hTERT cells lost viability (mid passage and late passage for the two donors), whereas BMI‐1 cells and in particular those containing the CMV promoter, maintained this elevated viability. In order to test the limit of our system, we also grew the cells further to infection passage 17/actual passage 20. Only the BMI‐1‐engineered cells reached this extended passage as hTERT cells failed to grow. Importantly, the extended passage BMI‐1 cells had lost their plasticity as assessed by the ability to differentiate at ALI and did not adhere or grow well on the transwells and had therefore reached the end of their lifespan (data not shown). These data demonstrating delay of cell senescence following BMI‐1 introduction are in good agreement with previous work that showed that cell senescence is delayed to passage 21–24 as we have shown but not sufficient for immortalization (Fulcher et al. 2009). These data also identified a potential advantage of the pLVX‐CMV lentivirus system. The hTERT data is in contrast to a recent study that engineered primary airway epithelial cells to express hTERT and reported that these cells grew beyond 40 passages, however, it is difficult to make conclusions as this study only used cells from one donor (Walters et al. 2013). Over extended passage the BMI‐1‐expressing cells maintained epithelial marker expression (CK14), whereas this was more variable with the hTERT‐engineered cells. To date, no other study has evaluated changes in CK14 marker expression post BMI‐1 engineering, however, both BMI‐1/hTERT‐ and hTERT‐engineered cells demonstrated maintenance of an epithelial phenotype based on additional markers including KRT5 in hTERT cell populations (Walters et al. 2013).

An important finding of our current study was that BMI‐1‐expressing cells (early‐mid‐late up to passage 15) maintain cell plasticity and the ability to differentiate at air–liquid interface generating a pseudostratified layer composed of basal, goblet, and ciliated cells as observed by sectioning transwells. These data are novel and significantly extend data on BMI‐1/hTERT‐ or hTERT‐engineered cells that can also maintain this capacity (Fulcher et al. 2009; Walters et al. 2013). However, the hTERT cell populations generated in this study did not maintain this plasticity and showed limited differentiation in contrast to a recent report (Walters et al. 2013)**.** These data question the utility of hTERT alone and suggest BMI‐1 alone to be the preferred genetic tool to maintain plasticity in our study. Of note, several groups are developing combination approaches to prevent cell senescence in primary epithelial cells using hTERT, for example, hTERT/BMI‐1 can lead to cells that reach at least 38 population doublings (Fulcher et al. 2009) and hTERT/cyclin‐dependent kinase 4 (Cdk4) epithelial cells have shown >100 population doublings (Roig et al. 2010). However, in this study, we focused to the use of BMI‐1 alone to reduce transformation of the cells away from a diploid karyotype and to maintain plasticity.

In agreement with the potential utility of BMI‐1 cell populations, further measures of differentiation in the context of the air–liquid interface model, mainly goblet cell (MUC5AC), ciliated cell (beta tubulin) staining, development of TEER, and SEM showing apical cilia were all maintained in the late passage BMI‐1 cell populations but not in the hTERT cell populations.

Overall, these data show that for the first time BMI‐1‐expressing cells have an extended lifespan, albeit not extensive and suggest that passage 15 cells show very similar plasticity properties to wild‐type cells for these key outcomes. Of critical importance, compared to other methods that incorporate the use of hTERT, we did not observe any alterations in karyotype in BMI‐1‐expressing cells, whereas the hTERT cells (p8) had chromosomal abnormalities. These findings are in complete agreement to the recent study of hTERT‐engineered bronchial epithelial cell populations which identified chromosomal abnormalities as early as passage 9 (Walters et al. 2013)**.** Interestingly, analyses of passage 14/15 BMI‐1/hTERT failed to identify chromosomal abnormalities suggesting that BMI‐1 may be protective in this context (Fulcher et al. 2009).

This study has several strengths over previous reports such as including the evaluation of multiple BMI‐1 and hTERT vectors in parallel in multiple primary human donors. Similarly, the extensive characterization of these cells for cell plasticity in the context of the ALI model and for karyotype is novel. These data from four genetically modified cell populations show comparable effects of BMI‐1 elevation. However, we acknowledge that greater numbers of donors and technical replicates are required to provide more quantitative characterization of cell differentiation, for example, TEER. This represents a limitation of this study. We have previously identified a large degree of heterogeneity in both intra‐ and interdonor variation in the air–liquid interface model (Stewart et al. 2012b), even in the absence of genetic modification suggesting a substantial study is required involving large numbers of donors over many years to further define quantitative changes in cell plasticity using this model.

In conclusion, we provide an in‐depth characterization of the plasticity of human bronchial epithelial cells that have been engineered to express BMI‐1. These cells retain the plasticity observed in wild‐type low passage cells including the ability to differentiate at ALI that can be used to model the lining of the airways. These findings suggest these cells may show utility in many aspects of respiratory research including in defining airway epithelial abnormalities in diseases such as asthma, which has the potential to lead to therapeutic opportunities.

## Conflicts of Interest

No conflicts of interest, financial or otherwise, are declared by the authors.
